# Human rabies control in Lebanon: a call for action

**DOI:** 10.1017/S095026881800300X

**Published:** 2018-11-15

**Authors:** M. F. Kassir, T. El Zarif, G. Kassir, A. Berry, U. Musharrafieh, A. R. Bizri

**Affiliations:** 1 Faculty of Medicine, American University of Beirut, Beirut, Lebanon; 2 Faculty of Medicine, Lebanese University, Beirut, Lebanon; 3 Communicable Diseases Department, Ministry of Public Health, Beirut, Lebanon; 4 Department of Family Medicine, American University of Beirut Medical Center, Beirut, Lebanon; 5 Department of Internal Medicine, Division of Infectious Diseases, American University of Beirut Medical Center, Beirut, Lebanon

**Keywords:** Estimating, prevalence of disease, prevention, public-health emerging infections, rabies (human)

## Abstract

The status of rabies as a neglected disease has made its eradication rather challenging in different parts of the world despite the availability of a successful vaccine. Lebanon, in particular, is a country endemic to the disease with several cases of rabies deaths reported over the past 30 years. The risk of rabies, however, has taken a new turn over the past few years in Lebanon with two emerging situations that have made the control of the disease rather challenging: the neighbouring Syrian war and the local garbage crisis. Both of these milestone events might have contributed to an increase in the number of disease vectors as well as individuals at risk, thus nourishing the cycle of disease transmission. In this observational study, the effect of these two events are investigated, with an update on the status of this preventable, yet often neglected, disease in the country. Both events were found to be concomitant with a notable increase in the number of dog bites and thus possible rabies exposure. Current regulations are explored through interviews with veterinarians, and custom recommendations, ranging from policies to control dog populations to awareness campaigns in high-risk individuals, are then proposed to help control the disease.

## Introduction

Rabies is a zoonotic infectious disease that has posed a significant health concern for humans since antiquity, with the oldest reports of the disease dating back to more than 4000 years ago [1]. Almost always a fatal disease of the nervous system [2], rabies used to be so terrifying that, across history, many would commit suicide at the mere possibility of having acquired this viral disease [1]. Fortunately, the introduction of a prophylactic rabies vaccine has rendered this disease 100% preventable [3], yet the status of the disease as ‘neglected’ has blocked its complete eradication as a health burden [4]. One of the main factors hampering this goal is the inadequate surveillance of the disease in many countries worldwide, which leaves our information on the spread, endemicity and burden of the disease incomplete [5]. However, despite the lacking information, rabies still accounts for more reported deaths than any other zoonotic disease worldwide, as current data show that it is responsible for almost 59 000 deaths globally per year [6, 7].

Most commonly, the rabies virus is transmitted by dog bites. Other wild animals might also serve as vectors for transmission, but infected dog bites still account for 95% of the total rabies-related deaths [2, 8]. Parenteral and oral vaccines are available to control the disease at the level of the vector itself, and these vaccines have been successfully employed in some high-income countries in which the disease was eliminated [9]. Various other dog population management tools such as surgical sterilisation and injectable contraceptives have also been employed to reduce the numbers of unmanaged dogs, and thus the feral vectors of the disease, but to varying levels of success [10].

In the Middle East, dogs are also the main vectors for transmission of rabies; the contribution of other animals such as cats and cattle is significantly less. In 2008, it was documented that almost 300 cases of human rabies are reported yearly from the Middle East region and that some countries including Saudi Arabia, Oman, Yemen, Israel, Iran and Turkey may face an escalating risk of wildlife rabies [11]. In a more recent report, the WHO has declared all of these countries endemic for dog rabies, in addition to Lebanon and Syria. Syria was considered endemic not only for dog rabies, but also for human dog-transmitted rabies [12].

Concerning Lebanon, two reports by our group have previously delineated the status of rabies in the country: one in 2000 and the other in 2013. A significant increase in the number of reported dog bites is noted when the two studies are compared. These studies pinpointed the potential relation between rabies in Lebanon and surrounding countries, especially with the porous borders that allow for the spillover of rabid dogs and other animals from Syria and Israel, both endemic with the disease [13, 14]. The aim of this report is to reflect the updated status of rabies in Lebanon, especially in light of two milestone events that might have significantly altered the situation of the disease: (1) the Syrian conflict and the resulting influx of Syrian refugees [15] and (2) the Lebanese national waste crisis [16]. The protracted Syrian conflict which began in 2011 has had a significant impact on the healthcare system in Lebanon, such as the emergence of an unprecedented number of leishmaniasis cases among the Syrian refugees in Lebanon [17, 18]. This makes it essential to measure the impact of the Syrian crisis on the status of other diseases such as rabies, especially that Hatch *et al*. have previously shown that the destruction of a country's internal infrastructure due to conflict can lead to a significant increase in the burden of canine-transmitted rabies [19]. On the other hand, the waste crisis might have played a role in amplifying the problem of stray dogs in Lebanon [20], which may increase the risk of human exposure to dog bites.

This paper will also delineate the status of the disease in the country from the perspective of experts in animal rabies. In order to achieve that, veterinarians from all around the country will be interviewed, in attempt to not only garner the experts’ opinion on the matter, but also investigate the measures and procedures followed by those experts to protect themselves as well as the community in which they live. This will serve as an update to the investigations previously carried out in our report in 2000 [13], besides setting the ground for more directed recommendations that might help curb a disease outbreak.

The impact of the two mentioned crises on the status of rabies in the country will hereby be studied, in attempt to propose feasible recommendations that are appropriate to contain any added burden of this unrelenting infectious disease.

## Methodology

### Rabies and animal bite record collection

Records on reported rabies cases, as well as dog bites, were collected from the Lebanese Ministry of Public Health (LMOPH) Epidemiological Surveillance Unit public database, which anonymously keeps track and reports these cases. Reported rabies cases, their dates, district, age and gender of the affected were collected starting 2013. Of interest to us were the records after 2013, since earlier cases were previously evaluated in our two previous reports on the subject [13, 14]. As for the dog bites, those reported in 2013–2016 were also collected from the LMOPH Epidemiological Surveillance Unit. The locations of the bite reports as well as the culprit animals were noted. Animal bites before this period were explored in our earlier reports [13, 14]. Data for 2017 were not made available by the Epidemiological Surveillance Unit. Moreover, the number of administered anti-rabies vaccines (Verorab) was collected from the records of the LMOPH in order to study the adequacy of the response to possible rabies exposure in the form of post-exposure prophylaxis; the number of vaccines administered per bite was then calculated and compared with the recommended post-exposure number of injections.

The LMOPH defines the confirmed rabies cases as those that display the paralytic or hyperactive symptoms of rabies and have been confirmed either by the detection of rabies viral antigens by direct fluorescent antibodies (either post-mortem in brain tissues or antemortem on skin or corneal smears), or by the detection of rabies-neutralising antibodies in cerebrospinal fluid of unvaccinated individuals. A rabies investigation form which includes the list of symptoms and the laboratory results is submitted by each centre to the LMOPH upon encountering a rabies case. The data on possible rabies exposure (animal bites) were also collected from anti-rabies centres, which submit a rabies exposure form to the LMOPH to document the cases of bites by possibly rabid animals.

### Working definitions

The status of rabies in Lebanon is described in this paper in light of two major events that have burdened the country: the garbage crisis and the neighbouring Syrian conflicts. The garbage crisis refers to the piling of garbage in Lebanese residential areas and streets starting July 2015; while industrial areas such as Dawra and Dekwaneh in the capital Beirut were particularly affected, satellite landfills and incineration sites were dispersed across the country [21]. This crisis was not properly dealt with and its impact could still be felt up until August 2016 and beyond [16]. As for the Syrian conflicts, they are defined by the protracted events that followed the trigger of the conflicts in 2011. These conflicts have led to a documented breakdown in basic public-health needs and infrastructure in the country [22].

### Interviewing the veterinarians

In order to assess the experts’ awareness and acknowledgement of the current status of rabies in Lebanon, interviews were held via phone calls with veterinarians registered in the Lebanese Order of Veterinary Physicians. Out of the 264 registered veterinarians, 120 were chosen randomly and then contacted. Out of those only 46 veterinarians were available for interviews, while the rest were either unavailable or unreachable. The veterinarians were asked to answer three main yes/no questions. The posed questions are the following:

1. Have you ever been vaccinated against rabies? If yes, was that as pre-exposure or post-exposure prophylaxis?
2. Do you recommend vaccinating all dogs presenting to your clinic?
3. Do you consider rabies to be a serious problem in Lebanon?

### Statistical analysis

Collected data on animal bites in the period following 2013 were compared with all available data on dog bites prior to this period – the data from 1991 to 1996 inclusive [13], as well as that from 2001 to 2012 inclusive [14]. The means of annual animal bites in these two periods were compared using an independent sample *t* test.

## Results

In our previous reports, the number of animal bites reported to the LMOPH were described for the period between 1991 and 1996 [13], as well as for the more recent span from 2001 to 2012 [14]. These values, along with the updated number of bites for 2013–2016 are presented in Table 1. Between 2005 and 2016, a total of 7369 animal bites were reported to the LMOPH with an annual average of 614 bites per year. Domestic and stray dogs, as well as cats, bats and rodents are amongst the culprit animals behind these bites. However, the major offending animal responsible for around 91% of all bites were dogs, with domestic dogs responsible for 53% of all bites. A remarkable increase in the number of bites by cats, rodents and other animals (27%) was noted in 2006.

**Table 1.** Animal bites in Lebanon as reported by the LMOPH Epidemiological Surveillance Unit

	Bites				Vaccine (Verorab)^a^	
	Domestic dogs	Stray dogs	Other animals^b^	Total	Total doses distributed	Doses per bite
2005	304 (61%)	140 (28%)	51 (10%)	495	1168	2.4
2006	321 (67%)	29 (6%)	131 (27%)	481	1570	3.3
2007	224 (57%)	133 (34%)	35 (9%)	392	1170	3
2008	261 (52%)	211 (42%)	30 (6%)	502	1265	2.5
2009	261 (53%)	213 (43%)	20 (4%)	494	1780	3.6
2010	188 (50%)	165 (44%)	26 (7%)	379	847	2.2
2011	175 (52%)	143 (42%)	21 (6%)	339	1223	3.6
2012	125 (46%)	133 (49%)	13 (5%)	271	1421	5.2
2013	365 (51%)	268 (38%)	76 (11%)	709	2557	3.6
2014	438 (52%)	335 (40%)	67 (8%)	840	3114	3.7
2015	580 (48%)	520 (43%)	96 (8%)	1196	3825	3.2
2016	692 (54%)	498 (39%)	81 (6%)	1271	4378	3.4
Total	3934	2788	647	7369	24 318	N/A
Annual average	328	232	54	614	2027	3.3

a Verorab™: purified vero-cell rabies vaccine.

b Cats, wild animals, bats, rodents and others.

Modified from the LMOPH website (http://www.moph.gov.lb).

**Fig. 1.**
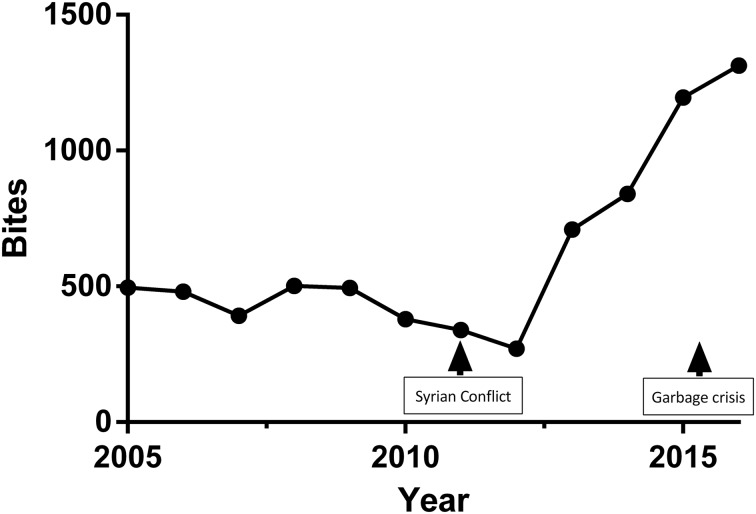
Total number of reported bites per year in Lebanon.

Between 2005 and 2013, the reported numbers of bites show a relative stability ranging between 271 and 502. In 2013, a steep increase in numbers was observed and was sustainable up to 2016 (Fig. 1 and Table 1). The yearly average of animal bites post-2013 is 1004 ± 272 bites per year, which is significantly greater than the average of 355 ± 145 bites per year prior to 2013 (span from 1991 to 1996 and 2001 to 2013), with a *P*-value = 0.014.

**Fig. 2.**
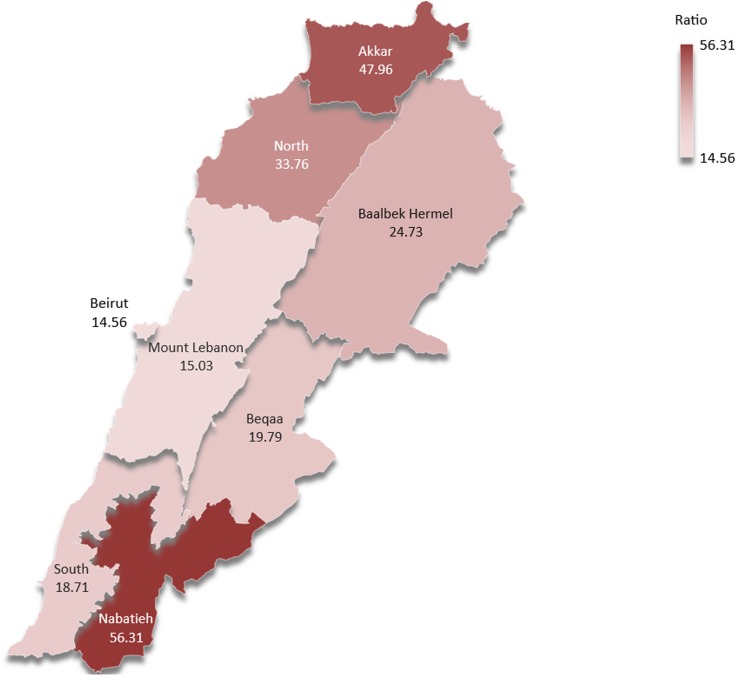
Bites/100 000 individuals in the different governorates of Lebanon in 2016 (map created using Microsoft Excel 2016 Map Chart tool – powered by Bing © Navteq).

The North and Nabatieh governorates had the highest number of dog bites per 100 000 individuals in 2016 as shown in Figure 2. With a bite rate of 56.31 and 47.86 bites/100 000 individual in Nabatieh and Akkar, respectively, these two governorates had a number of bites greater than the national average of 28.86 ± 15.8 bites/100 000 individual. It is worth noting that both Nabatieh and Akkar are governorates bordering Israel and Syria, respectively.

**Fig. 3.**
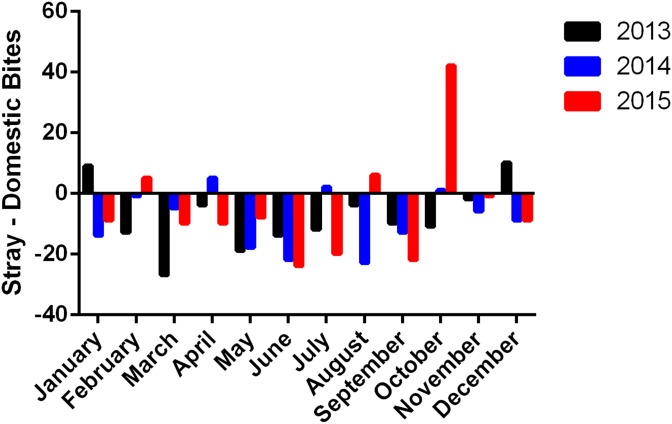
Difference between stray and domestic dog bites per month during 2013–2015.

**Fig. 4.**
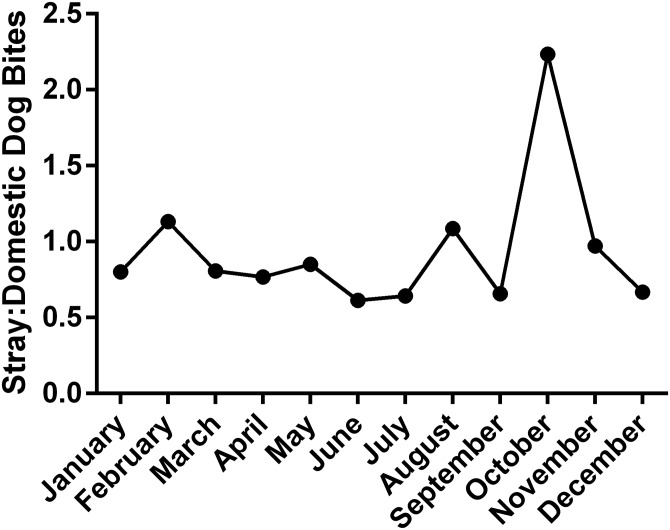
Ratio of stray to domestic dog bites in Lebanon in 2015.

The difference between the monthly distribution of bites between stray and domestic dogs throughout 2013–2015 was calculated and presented in Figure 3. While bites by domestic dogs were more prominent, records from October 2015 present a remarkable exception. With 42 more stray dog bites than domestic bites during this month, October of 2015 shows a difference in favour of stray dog bites significantly greater than the average monthly difference of −7 ± 12.81. This is also reflected by the peak in the stray to domestic dog bite ratio in October 2015 (Fig. 4).

In the period between 2013 and 2017, two cases of human rabies were detected and reported to the LMOPH in Lebanon (Table 2). Both those affected were Syrians, and one of them presented in Mount Lebanon while the other presented in the North. Prior to 2013, the nationalities of the incident rabies cases were not reported.

**Table 2.** Reported rabies cases in Lebanon

Year reported	2010	2010	2012	2013	2017
Sex	Male	Female	Male	Male	Male
Region	North	Bekaa	Beirut	Mt Lebanon	North^a^
Age group, years	20–39	60+	40–59	60+	5–9
Nationality	Unknown	Unknown	Unknown	Syrian	Syrian

a In the LMOPH records, the ‘North’ includes both the governorates of North and Akkar. Modified from the LMOPH website (www.moph.gov.lb).

The total number of vaccine doses provided by the LMOPH is also reported in Table 1. Vaccine doses of 3.3 were administered on average per animal bite during this period. These vaccines were not equally administered in different governorates. Although Beirut and Mount Lebanon witnessed about only 30% of the total bites in Lebanon between 2013 and 2016, Figure 5 shows that vaccinations are always higher in Beirut and Mount Lebanon compared with other governorates.

**Fig. 5.**
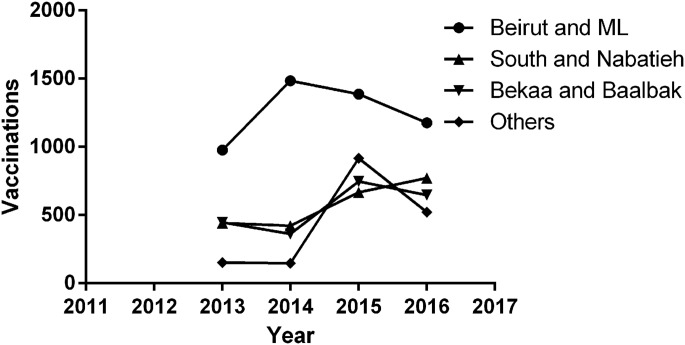
Vaccination records per governorate during the period between 2013 and 2016.

**Fig. 6.**
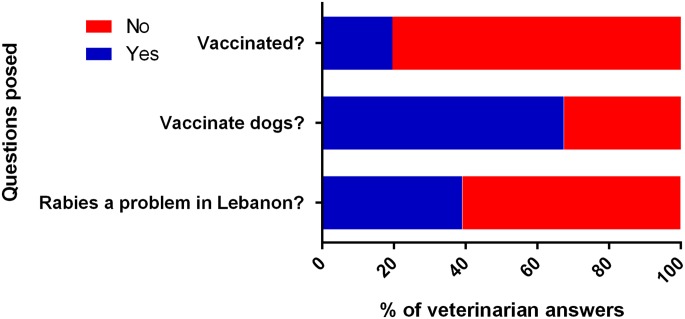
Answers of interviewed veterinarians to the posed questions.

The responses of the interviewed veterinarians are reported in Figure 6. All of the questions were categorical yes/no questions, and the percentages of recorded answers are displayed. The majority of interviewed veterinarians (80.44%) were *not* vaccinated against rabies. Of those vaccinated, only six were vaccinated as a form of pre-exposure prophylaxis by virtue of their constant occupational exposure to the virus. Most of the veterinarians (67.39%) stressed that no dog leaves their clinic without getting vaccinated against rabies, while the rest either did not have the vaccine available in their clinic, or left the choice of vaccination to the dog owners. Finally, while many of the respondents did not regard rabies as a pressing issue in Lebanon, still a considerable portion (39.10%) regarded rabies as a common finding in animals that must be monitored and controlled.

## Discussion

The available data reveal that after the last investigation on rabies in Lebanon [14], and more specifically between 2013 and 2016 inclusive, the average annual number of dog bites in the country was 1004, with the number of bites increasing from 709 in 2013 to 1271 in 2016 (Table 1). This average is significantly greater than the average number of bites in all the years investigated in previous reports [13, 14]; this is apparent in the sharp rise seen in Figure 1 which can be attributed to multiple factors such as an improvement in the reporting system. However, no changes in this system were announced by the LMOPH, and it is rather unlikely that the approximate doubling of the number of bites from 2010–2012 to 2013 be only due to better reporting. This general rise almost perfectly coincided with two impactful events in Lebanon and the neighbouring countries: the Syrian crisis of 2011 [15] and the Lebanese garbage crisis of 2015 [16].

The two reported rabies cases in Lebanon (Table 2) might have been patients who acquired the infection in Syria and sought healthcare in Lebanon, or else they could have caught the infection in Lebanon. The latter is very probable given the generally poor living conditions of Syrian refugees in Lebanon [23], which has predisposed them to the transmission of multiple communicable diseases [24]; in this case, their living conditions might have made them more prone to bites by stray animals, and this might have contributed to the sharp increase in dog bites in Lebanon since 2013. Another possible effect of war on the status of rabies might have been observed during the Lebanese–Israeli war in 2006, as a remarkably higher number of bites by cats, wild animals, rodents and bats was noted during that year. This might be attributed to the mass displacement of almost a third of the Lebanese population during this war [25], and thus the possible increase in exposure to bites by such animals which may be vectors for rabies.

The Syrian dog population, in fact, probably plays a big role in the rabies scene in Lebanon along with the Israeli dog population, as these neighbouring countries are both endemic for dog rabies [12]. The porous Lebanese borders with these countries mean that infected individuals, as well as virus-carrying dogs, might easily cross from one country to the other [26]. The distribution of bites across the Lebanese governorates shows clearly increased incidence of bites in Akkar and Nabatieh which are border governorates (Akkar with Syria and Nabatieh with Israel). This proximity with countries endemic for the disease makes residents of the governorates more susceptible to the infection with the added burden of dog-bites by vectors crossing the borders [26]. This is noteworthy as a study conducted in Sierra Leon revealed that the incidence of urban canine-transmitted rabies increases significantly in times of war, especially with the destruction of a country's healthcare infrastructure [19]. This is comparable with the situation in Syria since 2011, where healthcare services and facilities have been destroyed, which might have led to an exacerbation of the rabies endemicity, as in Sierra Leon [22]. Therefore, it is expected that the aggravation of this infectious disease would lead to more rabid vectors, which can easily pass across the unmonitored borders onto Lebanese grounds.

The other perpetrator possibly responsible for this abrupt increase in the number of dog bites after 2013 is the garbage crisis which has recently faced Lebanon [16]. The accumulation of wastes in dumpsites led to the declaration of a severe problem in July 2015, and these open garbage dump sites have been previously shown to contribute to the rise in the number of stray dogs which amplifies the number of possible vectors [20]. Garbage dumps are breeding areas of stray dogs, and if they are no longer around, dogs will migrate to other places. This is reflected by the peak in the stray to domestic dog ratio in October 2015 (Fig. 4), after heaps of garbage had been covering the Lebanese streets for several months [16]. October, in fact, witnesses the beginning of the rain season in Lebanon [27], and the rainfall in the presence of open garbage dumps leads to the formation of leachate, a polluting by-product of organic matter. This poses both social and environmental problems such as nuisance, diseases and the spread of stray dogs and other harmful animals [28]. This rise in stray dogs increases the possibility both of new vectors as well as new bites. It is noteworthy that this predominance of stray dog bites was only observed in October 2015, while it was not present in either 2013 or 2014 (Fig. 3). This further strengthens the correlation between the garbage crisis, a special circumstance of October 2015, and the increase in stray dog bites.

As for the vaccines, the Center for Disease Control and Prevention (CDC) recommends that a regimen of four 1-ml doses of Human Diploid Cell Vaccine/Purified Chick Embryo Cell (HDCV/PCEC) vaccines and Rabies Immunoglobulins (RIGs) be administered intramuscularly to previously unvaccinated persons on days 0, 3, 7 and 14 after exposure. Immunocompromised persons might be candidates for a 5th dose on day 28. Previously immunised individuals, on the other hand, must receive post-exposure prophylaxis in the form of a regimen of two 1-ml doses of HDCV or PCEC vaccines without RIGs administered intramuscularly on days 0 and 3 post-exposure [29]. The annual average of rabies vaccine doses/bite, however, is 3.3 and is thus yet to reach the four-dose goal. Administering fewer doses might either be due to patients lost to follow-up, or to cases in which the culprit animal is known not to be rabid (rarely is the animal followed) [13]. Figure 5 shows that more vaccines were administered in Beirut although the number of bites in Beirut is less than that of other governorates which is consistent with the role of the hospitals of Beirut, the capital of Lebanon, as reference centres attracting patients from all the different governorates.

The discussed data are, of course, not free of potential sources of error and uncertainty. Reporting of both dog bites and infections is not a straightforward process and is prone to error at different stages. The patients must be educated enough to recognise any animal bite as a serious threat. Only then would they reach out to a nearby physician or hospital, which is a potential problem in rural areas where the disease is more prevalent, as was discussed earlier [30]. Besides, the LMOPH has established a protocol that requires physicians to report possible cases of communicable diseases to district authorities, and this will depend on the competency of both the physicians and the authorities. The shortages of the reporting system are further highlighted by the fact that as of the date of submission of this paper, the most recent reports on the number of dog bites in Lebanon date back to 2016.

Interviewing the veterinarians further revealed the general nonchalance towards this deadly disease in Lebanon. A very small percentage of the interviewed veterinarians were vaccinated against rabies as a prophylactic measure given the possibility of exposure to the virus in any of the animals presenting to their clinic. The CDC and American Veterinary Medical Association both strongly recommend that *all* veterinarians be vaccinated prophylactically against rabies, given their status as a frequent-risk group in regard to the disease [31, 32]; the veterinarians in Lebanon are clearly still short of complying with these recommendations. In a survey conducted by Trevejo to measure rabies pre-exposure vaccination rate and identify factors potentially associated with lack of vaccination among veterinarians and at-risk staff, the vaccination rate was high among veterinarians, although follow-up with recommended serologic testing and boosters was low [33]. Other at-risk staff had much lower vaccination rate due to the lack of immunisation regulatory requirement as is the case with the Lebanese veterinarians [33].

While most of the interviewed veterinarians insisted on vaccinating all dogs presenting to the clinics, some still believed the dog-owners can choose not to vaccinate their pets. Given the fact that most reported dog bites are in fact by domestic dogs (Table 1), this raises the question of whether the current level of protection against the disease is adequate. Veterinarians, in general, can fully support a pet owner's refusal to vaccinate their pet with a comfortable leeway for discussion; this is unlike immunisation in humans, as paediatricians, for example, take a very firm stance against parents who refuse to vaccinate their children as revealed by a survey conducted by the American Academy of Pediatrics in 2005 [34]. This generally lax attitude towards the disease was finally reflected in the veterinarians’ answers to our last question; the majority did not regard the disease as a problem in Lebanon, despite the fact that Lebanon has been officially labelled by the WHO as endemic for animal rabies [12], and despite the other factors discussed in this paper.

In spite of all what has been discussed, it is still reasonable to believe that the Lebanese healthcare system will show a sturdy resilience in its coping to any additional burden of rabies, as it has with other recent health problems [18]. However, it is advisable that a clear plan be devised to limit any surge in the incidence of the disease and thus avoid having to deal with preventable repercussions. The first step along this path should be to have high-risk individuals such as veterinarians, dog owners, hikers, farmers and members of the army understand the potential threat of getting bitten by a rabid dog and consider pre-exposure prophylaxis. The Lebanese Order of Veterinary Physicians should emulate the American Veterinary Medical Association in their compliance to the CDC recommendations regarding the necessity of vaccination in veterinarians.

In addition to increasing public awareness, a strategy must be set to control dog populations properly, especially that dogs constitute the major vectors of the disease in the region [11]. Many available practices could be the cornerstone of this strategy, such as controlling stray dog breeding through the use of contraceptives, sterilisation, confinement and ultimately euthanasia [10]. We may extrapolate from the experiences of more endemic countries such as Sri Lanka, where a ‘no kill’ approach to roaming dogs with a ‘capture, neuter, vaccinate and release’ policy was successful [35]. The contribution of domestic dogs to the burden of rabies can also be limited by legislative action. Responsible Dog Ownership guidelines can be officially set to formally elucidate the responsibilities of every dog owner. The dog owners should be held responsible not only for the provision of food, water and shelter for their pets, but also for the vaccination of those pets against diseases such as rabies and thus the protection of the pets’ health [10]. Vaccinating dogs is a powerful and essential public-health intervention to break the transmission cycle. A penalty system must be constructed to ensure compliance to these laws. Mandatory microchipping of domestic dogs can facilitate this process, as it helps the authorities keep track of every dog's vaccination records. This practice has also proven successful in reuniting lost dogs with their owners [36], which might be of immense importance in the control of the stray dog population. It might also save governmental funds that would be otherwise spent on the aforementioned stray dog population management tools.

The main goal, indeed, should be proper garbage management, which will not only decrease the burden of stray dogs, but also limit the many other adverse health effects of unplanned dumpsites. Controlling the dog populations might however not be enough, especially with the possible influx of infected dogs from neighbouring countries. At the public level, education and awareness programmes must be put in place to inform the population on dog bite prevention and treatment [37]. These programmes should focus their efforts on addressing the populations with the highest risks, namely the Syrian refugees living in camps as well as the residents of rural areas. Last but not least, post-exposure prophylaxis vaccines must be made available in public-health centres and referral centres in all regions in Lebanon free-of-charge for cases when prevention fails.

## Conclusion

The rabies problem remains a concern in Lebanon aggravated by the on-going circumstantial burdens of both the Syrian war and garbage waste crisis. The approach to this problem should be attained at more than one level: develop a national strategy for elimination of dog-mediated rabies that ensures a systematic management of susceptible animals and humans under the relevant laws and regulations. Rabies in humans can be prevented by eliminating exposures to rabid animals and by pre-/post-exposure vaccination. Continuous education of health professionals on proper dog bite management, and administration of post-exposure prophylaxis is necessary to provide effective prevention of human rabies. On the other hand, effective rabies surveillance in humans and animals enhances early detection and reporting of cases and is important for initiating timely responses and enabling informed decisions about when and where to intensify rabies control efforts. Under the national strategy other important initiatives need to be implemented and these include: awareness promotion programmes aimed at increasing attentiveness to the disease among the public; activating the role of municipalities in containing stray dogs and scaling up their vaccination and enforcing responsible ownership with penalties for those not abiding by proper pet care and treatment and the provision of vaccines and pre- and post-exposure prophylaxis by the LMOPH when needed. Specific to Lebanon are two related aggravating issues: the current garbage crisis that unless adequately managed will be a driving catalyst to the rabies spread in Lebanon and the porous borders that need to be controlled in order to restrict the influx of stray dogs that have the capacity to cross boundaries. In this respect, regional cooperation is emphasised.
