# Human rabies control in Lebanon: a call for action – CORRIGENDUM

**DOI:** 10.1017/S0950268821000352

**Published:** 2021-02-26

**Authors:** M. F. Kassir, T. El Zarif, G. Kassir, A. Berry, U. Musharrafieh, A. R. Bizri

**Affiliations:** 1 Faculty of Medicine, American University of Beirut, Beirut, Lebanon; 2 Faculty of Medicine, Lebanese University, Beirut, Lebanon; 3 Communicable Diseases Department, Ministry of Public Health, Beirut, Lebanon; 4 Departmentof Family Medicine, American University of Beirut Medical Center, Beirut, Lebanon; 5 Department of Internal Medicine, Division of Infectious Diseases, American University of Beirut Medical Center, Beirut, Lebanon

The original publication of this article contained incorrectly cited paper in the reference list that omitted the authors’ names from the citation. The affected citation is number 28 in the reference list and the correct citation is shown below.

**El-Kelani, R., Shadeed S., Hasan, A., Ghodieh, A., Burqan, M.** (2017) Geospatial Implications Assessment of Zahrat Al Finjan Solid Waste Landfill, North of West Bank, Palestine, IUG Journal of Natural Studies Vol. 25, No 2, 1–9.

This error has now been corrected in the original online publication.
